# Can Brain Volume-Driven Characteristic Features Predict the Response of Alzheimer’s Patients to Repetitive Transcranial Magnetic Stimulation? A Pilot Study

**DOI:** 10.3390/brainsci14030226

**Published:** 2024-02-28

**Authors:** Chandan Saha, Chase R. Figley, Brian Lithgow, Paul B. Fitzgerald, Lisa Koski, Behzad Mansouri, Neda Anssari, Xikui Wang, Zahra Moussavi

**Affiliations:** 1 Biomedical Engineering Program, University of Manitoba, Winnipeg, MB R3T 5V6, Canada; 2 Department of Radiology, University of Manitoba, Winnipeg, MB R3T 2N2, Canada; 3 Department of Psychiatry (MAPRC), Monash University, Melbourne VIC 3004, Australia; 4 Department of Psychology, Faculty of Science, McGill University, Montreal, QC H3A 1G1, Canada; 5 Brain, Vision and Concussion Clinic-iScope, Winnipeg, MB R2M 2X9, Canada; 6 Warren Center for Actuarial Studies and Research, University of Manitoba, Winnipeg, MB R3T 5V4, Canada

**Keywords:** Alzheimer’s disease (AD), rTMS treatment, DLPFC, MRI analysis, efficacy prediction

## Abstract

This study is a post-hoc examination of baseline MRI data from a clinical trial investigating the efficacy of repetitive transcranial magnetic stimulation (rTMS) as a treatment for patients with mild–moderate Alzheimer’s disease (AD). Herein, we investigated whether the analysis of baseline MRI data could predict the response of patients to rTMS treatment. Whole-brain T1-weighted MRI scans of 75 participants collected at baseline were analyzed. The analyses were run on the gray matter (GM) and white matter (WM) of the left and right dorsolateral prefrontal cortex (DLPFC), as that was the rTMS application site. The primary outcome measure was the Alzheimer’s disease assessment scale—cognitive subscale (ADAS-Cog). The response to treatment was determined based on ADAS-Cog scores and secondary outcome measures. The analysis of covariance showed that responders to active treatment had a significantly lower baseline GM volume in the right DLPFC and a higher GM asymmetry index in the DLPFC region compared to those in non-responders. Logistic regression with a repeated five-fold cross-validated analysis using the MRI-driven features of the initial 75 participants provided a mean accuracy of 0.69 and an area under the receiver operating characteristic curve of 0.74 for separating responders and non-responders. The results suggest that GM volume or asymmetry in the target area of active rTMS treatment (DLPFC region in this study) may be a weak predictor of rTMS treatment efficacy. These results need more data to draw more robust conclusions.

## 1. Introduction

Repetitive transcranial magnetic stimulation (rTMS) has been investigated as a treatment for Alzheimer’s disease (AD) in the last decade. Several rTMS studies have reported its effectiveness for AD treatment [1,2,3,4]. However, the treatment protocols of rTMS for AD are demanding for the families and patients as they usually involve 2–6 weeks of daily treatment [5,6], and some also continue maintenance treatment for up to 6 months [4,7]. However, not everyone responds positively to rTMS treatment. Furthermore, in our recent large clinical trial [8], we observed that a number of patients declined after rTMS treatment. Therefore, there is uncertainty regarding the efficacy of rTMS. Given that rTMS is an expensive and resource-intensive technology with a demanding treatment protocol for patients, the ability to predict a patient’s response before the commencement of rTMS would be very beneficial and could lead to the development of a more individualized therapeutic strategy.

In this study, we analyzed magnetic resonance imaging (MRI) images of participants with AD obtained at baseline in a recent clinical trial [8] to investigate whether brain volume estimates have the potential to predict the patient’s response to rTMS treatment. MRI is commonly used to examine gray matter (GM) and white matter (WM) volume anomalies in AD brains [9,10]. In addition, its potential to predict tissue loss in distinct brain regions has been reported in dementia studies [11,12]. Another application of MRI in rTMS clinical trials, including in [13], is for the neuronavigation of the magnetic coil during treatment, in which MRI scans of AD participants are utilized to localize a target area of the brain, in the case of our study, to target stimulation to the dorsolateral prefrontal cortex (DLPFC) bilaterally. The DLPFC is the most common brain region for the treatment of AD using rTMS [14] due to its broad and complex connections with cortical and deeper subcortical brain structures [15] and its executive role in planning and decision -making, most notably in working memory [16,17]. However, to date, no study has investigated the potential of baseline MRI analysis to predict rTMS treatment efficacy in the AD population.

The left and right DLPFC were the target sites of rTMS intervention for AD participants in our clinical trial [8]; thus, in this study, we investigated whether the volume of the DLPFC region estimated from the baseline MRI scans differed between responders and non-responders to rTMS treatment. We investigated this hypothesis in AD participants undergoing rTMS treatment separately by measuring (1) the GM or WM volume of each side of the DLPFC and (2) the magnitude of the asymmetry index of the DLPFC from its GM or WM volume. In addition, we explored baseline structural differences between responders and non-responders in other brain regions using whole-brain analysis. Furthermore, we examined whether the abovementioned DLPFC measures correlated with baseline cognitive scores.

## 2. Materials and Methods

### 2.1. Participants

Initially, 128 participants with AD from our rTMS clinical trial [8] who had MRI data at baseline and completed either active (*n* = 86) or sham (*n* = 42) rTMS treatment with follow-up post-treatment assessments were included. Subsequently, 18 subjects were excluded owing to inadequate image quality and different MRI scanning parameters; the remaining 110 participants’ MRIs (75 in the active group and 35 in the sham group) were included. All participants with AD and their primary caregivers provided written consent prior to enrollment in the study, which was approved by the local ethics committee at each site of the rTMS treatment study (Winnipeg, Montreal, and Melbourne) [17]. The diagnosis of AD was made by a neurologist or neuropsychiatrist based on the participants’ clinical history and neuroimaging results from MRI and/or fluorodeoxyglucose positron emission tomography (PET) scans. Using the Super Rapid-2 Magstim system (manufactured by the Magstim Company Limited, Spring Gardens, Whitland, UK), the protocol of rTMS application was to deliver 25 1.5 s trains of pulses at 20 Hz with an intertrain interval of 10 s applied to the DLPFC bilaterally (750 pulses to each side, serially) for either 2 or 4 consecutive weeks (5 days/week) [8]. The pulses were neuronavigated using the MRI scan of each participant and were applied at 100% of the resting motor threshold of each participant. The protocol of sham and active stimulations was exactly the same.

The primary outcome measure of the clinical trial [8] was the Alzheimer’s disease assessment scale—cognitive subscale (ADAS-Cog), and the secondary outcome measures were the Neuropsychiatric Inventory–Questionnaire (NPI-Q) and Alzheimer’s Disease Cooperative Study—Activities of Daily Living Inventory (ADCS-ADL) to evaluate rTMS treatment efficacy. Each participant’s response to rTMS was measured by comparing the baseline ADAS-Cog, NPI-Q, and ADCS-ADL scores with those at either week 5 or post-treatment after week-8 assessments, as detailed in [13]. In brief, a marked response is defined as an ADAS-Cog improvement with a score of 3+. A moderate response is referred to as an improvement (<3 scores) in ADAS-Cog **AND** the same score **OR** improvement in ADCS-ADL **OR** NPI-Q scores. The response is considered small if the **AND** part does not hold in the previous moderate response criterion. A small/stabilized response is also defined as a non-substantial decline in ADAS-Cog (<3 score decline) **AND** an improvement in both ADCS-ADL and NPI-Q scores by 1. If the **AND** part does not meet the previous small/stabilized response criterion, it is considered non-responsive. Notably, in all response criteria mentioned above, the **AND** represents a Boolean logical AND operator (it is only “true” if both statements are true and otherwise “false”), and **OR** is a Boolean logical OR operator (it is “true” if either one of the statements or both statements is true and otherwise “false”).

In this study, we focused on predicting the response in the active rTMS group (*n* = 75), in which 42, 13, 10, and 10 participants had marked, moderate, small, and no responses, respectively. To perform a response group-wise comparison with a sufficiently large sample size, we combined them into binary response groups under active treatment: responders (participants with marked and moderate responses, *n* = 42 + 13 = 55) and non-responders (participants with small/stabilized and non-responses, *n* = 10 + 10 = 20).

### 2.2. MRI Data Acquisition

T1-weighted structural MRI scans were acquired using a 3D magnetization-prepared rapid acquisition gradient-echo (MPRAGE) imaging sequence. Our rTMS efficacy study on AD [17] was run at three different sites: Winnipeg (3T Siemens Verio/Verio Dot MRI system), Montreal (3T Siemens Prisma/Prisma-fit MRI system), and Melbourne (3T Siemens Skyra/Skyra-fit MRI system). The imaging sequence parameters from all sites were as follows: slice thickness = 0.9–1.2 mm, echo time = 2.22–2.98 ms, repetition time = 1800–2300 ms, inversion time = 900/1100 ms, and flip angle = 8–10 degree.

### 2.3. MRI Data Analysis

Structural MRI data were analyzed by Voxel-Based Morphometry (VBM) using the Computational Anatomy Toolbox (CAT12, v12.7, The Structural Brain Mapping Group, University of Jena, Germany, http://www.neuro.uni-jena.de/cat/, accessed on 3 May 2021) [18] and Statistical Parametric Mapping software (SPM12, v7771,The Wellcome Centre for Human Neuroimaging, University College, London, UK, https://www.fil.ion.ucl.ac.uk/spm/, accessed on 3 May 2021). A standard “unified segmentation” approach of SPM [19] segmented the denoised [20], bias field-corrected, and affine-registered T1-weighted MRI data into tissue maps of GM, WM, and cerebrospinal fluid (CSF). This segmentation was then passed through the refining process to attain the final stage of the adaptive maximum a posteriori segmentation [21]. Subsequently, the T1-weighted image and GM, WM, and CSF masks were non-linearly normalized to the Montreal Neurological Institute (MNI) template using geodesic shooting [22]. Simultaneously, CAT12 performed several automated quality assurance checks and estimated the total intracranial volume (TIV). Finally, the segmented images were modulated to control for the amount of deformation due to differences in brain size [23] and were used in the following region-of-interest (ROI) analysis and whole-brain voxel-wise comparisons.

**Figure 1:**
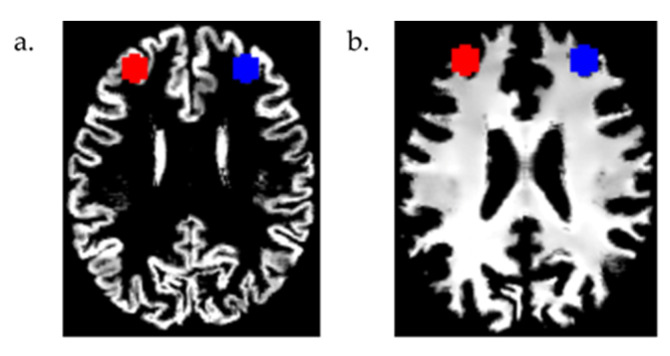
The left (red) and right (blue) dorsolateral prefrontal cortex (DLPFC) masks are shown on the modulated and warped (**a**) gray matter and (**b**) white matter images (axial view) of a participant.

Since the participants received rTMS treatments targeting both the left and right DLPFC, we created bilateral ROI masks using two 8 mm radius spheres centered at MNI coordinates x = 30, y = 43, and z = 23 (right DLPFC), and x = −30, y = 43, and z = 23 (left DLPFC) in the MarsBar [24] toolbox (v0.45, http://marsbar.sourceforge.net/, accessed on 28 March 2022). These MNI coordinates of the DLPFC, reported in previous studies [25,26], are slightly deeper than the Talairach coordinates (x = ±50, y = 30, and z = 36), in which the coil position and direction are specified using the BrainSight 2 software (Rogue Research, Montreal, QC, Canada) in the clinical trial of rTMS [17]. Instead of using these Talairach coordinates, because of their proximity to the skull, we used the MNI coordinates of the DLPFC [25,26] mentioned above to develop the two ROI masks. These masks were resliced, and the volumes from the modulated and warped GM and WM images were then calculated using the get_totals.m script by G. Ridgeway (http://www0.cs.ucl.ac.uk/staff/g.ridgway/vbm/get_totals.m, accessed on 1 April 2022). The overlays of these masks on a participant’s modulated and warped GM and WM images are shown in Figure 1 using the MRIcron [27] software (v1.0.20190902, University of South Carolina, Columbia, SC, USA, https://people.cas.sc.edu/rorden/mricron/, accessed on 15 August 2023).

Left and right asymmetries are cardinal features of the brain [28]. In this study, we investigated whether there was a difference in volumetric asymmetry between responder and non-responder groups. GM asymmetry using the VBM technique with T1-weighted MRI data has been widely studied [29]; however, WM asymmetry incorporating this method is less consistent [30]. Nevertheless, prior studies [9,10,31] have performed VBM on T1-weighed MRI data, and Good et al. [32] also used it for WM asymmetry analysis. This study analyzed left GM or WM volumetric asymmetry between bilateral DLPFC regions for each population, where the asymmetry index was calculated separately for GM and WM volumes, using the following formula [33,34]:

(1) $$Asymmetry\;index=\frac{\left|(left-right)\right|}{left+right}\;*\;100$$

Note that the raw volume estimates of the GM or WM were used to calculate the asymmetry index, and lower values indicate more symmetry (i.e., less asymmetry) between the bilateral DLPFC ROIs.

The extracted volumes and asymmetry indices of the responders and non-responders were statistically analyzed (described below). More exploratory post-hoc whole-brain comparisons were then conducted using group-wise (2nd level) statistical analysis in SPM12 to investigate whether any other brain regions (in addition to the bilateral DLPFC) might be useful for rTMS response prediction. To achieve this, the modulated and spatially normalized images (GM and WM) were smoothed using an 8 mm isotropic full width at half-maximum Gaussian kernel to account for potential differences in segmentation and non-linear normalization accuracy between participants. The 2nd level statistical analysis was then set up in SPM12 using a two-sample *t*-test with two contrasts (responders > non-responders and responders < non-responders) and participants’ age, sex, TIV, MRI site, and Cornell Scale for Depression in Dementia (CSDD) scores as covariates. GM and WM analyses were run separately, and family-wise error (FWE) in multiple comparisons was corrected to *p* < 0.05. The extent of the threshold, k > 50 voxels, was set to consider a cluster significant.

### 2.4. Statistical Analysis

A two-proportion test was used to examine statistical differences in baseline categorical data between the two response groups under active treatment. The independent samples *t*-test or Wilcoxon rank-sum test was also used depending on whether specific continuous data were normally distributed (checked by the Shapiro–Wilk test).

Analysis of covariance (ANCOVA) was employed to find differences in ROI data between responders and non-responders under active treatment. ANCOVA is a blended version of the analysis of variance and regression [35] that allows for the control of the influences of covariates, including age, sex, TIV, MRI site, and CSDD scores. GM or WM volume in each ROI was used as a dependent variable.

To further investigate the possible lateralization of the DLPFC region in responders and non-responders under active treatment, we performed a paired *t*-test to compare the normalized (divided by TIV) left and right volumes of GM and WM. Moreover, we used ANCOVA to compare the magnitude of the GM or WM asymmetry index in the DLPFC region between responders and non-responders. The asymmetry index of the GM or WM as a dependent variable and age, sex, TIV, MRI site, and CSDD scores as covariates were used to build the ANCOVA model. Statistical analysis was performed using the R platform after installing the required packages in RStudio (ver. 1.4.1106) [36,37]. To control for multiple comparisons across the two ROIs (left and right DLPFC), we employed Bonferroni correction to control for family-wise error (*p* < 0.05/2 = 0.025, significance threshold).

We employed logistic regression as a predictive classification model and evaluated its performance using a five-fold cross-validation with five repetitions. Additionally, we employed the synthetic minority oversampling technique (SMOTE) [38], as our two response groups’ sizes were imbalanced, and the predictive model might be biased toward the over-represented group, that is, responders. In SMOTE, new and non-replicated instances are generated in the minority group, whereas the conventional oversampling scheme has an overfitting issue [39].

Of the 35 participants in the sham group, 12 individuals (responders = 8 and non-responders = 4) received active treatment after the study period (6 months) in an open-label study. Their data were added to the active treatment group, and the analyses were repeated.

## 3. Results

### 3.1. Baseline Characteristics of Participants

Table 1 presents the demographic data of the study participants and baseline CSDD, Montreal Cognitive Assessment (MoCA), Clinical Dementia Rating (CDR), and ADAS-Cog scores of the active treatment group. Responders and non-responders did not show significant differences in sex, age, or handedness. These participants had no major depressive disorder, and there was no substantial difference in CSDD scores between the response groups. In cognitive scores, similarity was demonstrated in MoCA and CDR scores between responders and non-responders, while the responders had significantly higher ADAS-Cog scores (implying more cognitive impairment) than the non-responders.

### 3.2. Region of Interest (ROI) Analyses

#### 3.2.1. GM and WM Volume

After adjusting for covariates, the analysis of covariance showed that the responders in the active treatment group had significantly lower GM volume in the right DLPFC region (*p* = 0.004) than non-responders (Table 2). No significant differences were observed between responders and non-responders in the GM of the left DLPFC or in the WM of either the left or right DLPFC.

#### 3.2.2. Lateralization and Asymmetry Index

**Figure 2:**
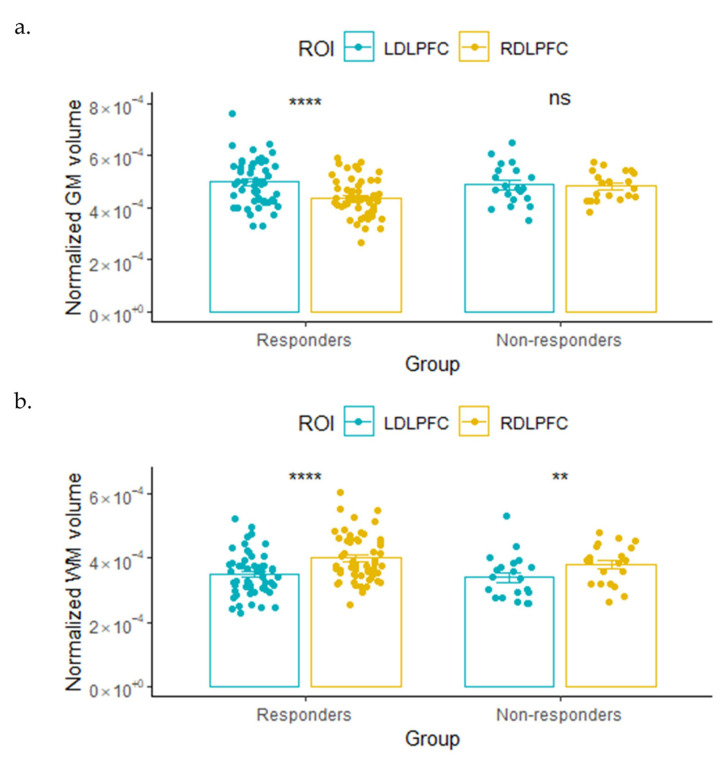
Comparison of normalized (**a**) gray matter (GM) or (**b**) white matter (WM) volumes (mean ± SE) between left DLPFC and right DLPFC for responders and non-responders under active treatment. Normalized volume is calculated by dividing each region’s raw volume of GM or WM by the total intracranial volume (TIV). Results of the two-tailed paired *t*-test are shown (**** *p* ≤ 0.0001, ** *p* ≤ 0.01, and ns: *p* > 0.05).

Responders under active treatment had significant leftward lateralization (left > right) in the GM volume and rightward lateralization (left < right) in the WM volume of the DLPFC region, as shown in Figure 2 (paired *t*-test). In contrast, non-responders only had significant rightward lateralization (left < right) in the WM volume of the DLPFC. No significant lateralization was observed in the GM volumes of non-responders in the active treatment group.

In the comparative analysis of the volumetric asymmetry index using ANCOVA, the responders showed a significantly higher GM volumetric asymmetry index (*p* = 0.009) in the DLPFC region compared to non-responders (Table 3). However, the asymmetry index in WM was not significantly different between the groups.

### 3.3. Predictive Classification Results

We assessed the performance of logistic regression for classifying responders and non-responders (75 participants) utilizing each significant GM feature of the ROI (GM volume of the right DLPFC and GM asymmetry index of the DLPFC) alone and their combinations (Table 4). As expected, when both GM features of the ROI were used in the logistic regression model, it provided the highest accuracy (0.69), with an area under the curve (AUC) of 0.74 for separating responders and non-responders receiving active treatment.

### 3.4. Whole-Brain Analysis Results

The exploratory whole-brain analysis of GM and WM volumes did not reveal any other areas with statistically significant differences between responders and non-responders in the active treatment group after accounting for age, sex, TIV, MRI site, and CSDD as covariates and applying FWE correction for multiple comparisons. Even the right DLPFC region, which demonstrated a significant outcome in the ROI analysis of participants under active treatment, did not survive FWE correction in the whole-brain analysis.

### 3.5. Correlations between ROI Volumes and Baseline ADAS-Cog Scores

**Figure 3:**
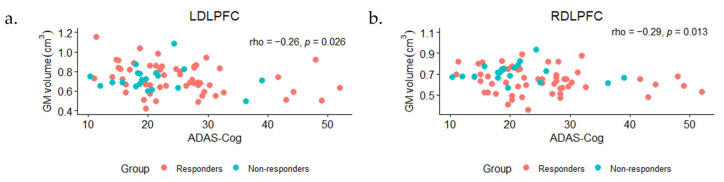
Scatter plots of baseline ADAS-Cog scores of responders and non-responders under active treatment and their raw GM volumes (cm^3^) of (**a**) left and (**b**) right dorsolateral prefrontal cortex (DLPFC).

Spearman’s correlation analysis for non-normally distributed data was computed between raw data points of GM volume in the ROIs of responders and non-responders and their baseline ADAS-Cog scores. As shown in Figure 3, a significant correlation was observed between baseline ADAS-Cog scores and GM volume in the left DLPFC (rho = −0.26, *p* = 0.026) and right DLPFC (rho = −0.29, *p* = 0.013). After controlling for age, sex, and TIV in the partial correlation analysis, GM volume in the right DLPFC of responders and non-responders showed a significant correlation (rho = −0.23, *p* = 0.048) with baseline ADAS-Cog scores.

### 3.6. Results after Adding the 12 Participants Who Received Active Treatment after the Sham 6–7 Months after the Baseline

After adding the baseline data of the 12 participants who received active treatment after sham treatment (~7 months after the baseline) to the initial active treatment group (now, *n* = 87), the ANCOVA showed significant differences [F (1, 79) = 5.55, *p* = 0.021] between responders and non-responders in the GM asymmetry index of the DLPFC.

## 4. Discussion

In this study, we investigated whether baseline structural brain MRI data could predict the efficacy of rTMS treatment for cognitive impairment in patients with mild-to-moderate AD. We herein estimated the GM or WM volumes and asymmetry index in the DLPFC region of the brain and compared those measures between responders and non-responders to rTMS treatment.

The main findings of this study were a significantly lower GM volume in the right DLPFC and higher GM asymmetry of the DLPFC among responders compared to non-responders under active treatment. The responders and non-responders under active treatment did not differ significantly in either CDR or MoCA scores; however, the ADAS-Cog score was significantly higher, representing worse cognitive performance in responders than in non-responders. An important point is that a “ceiling effect” could explain the results of this study. The responders had higher ADAS-Cog scores at baseline and more GM asymmetry in the DLPFC area. Therefore, it was easier to observe a benefit in them after rTMS. It was harder to see a benefit in the non-responders because of the “ceiling effect” (they had baseline ADAS-Cog scores closer to normal).

In general, GM atrophies in the DLPFC areas [40] and GM asymmetry [29] have been reported to be associated with AD. In this study, we focused on the rTMS treatment target area, that is, the DLPFC, and it is possible that in comparison to non-responders, the responders to active treatment at baseline might have been more affected in the pathogenesis of amyloid-beta, tau tangles, and neurodegeneration, particularly in the areas of the DLFPC. We also speculate that the response to treatment is affected by the presence of GM asymmetry in the DLPFC, caused by the neuropathology of AD. Iaccarino et al. [41] reported that amyloid beta accumulates in the association cortex (surrounding sensory and motor regions) in the early stage of dementia, and the distribution of tau tangles may extend up to the lateral occipital and areas of the DLPFC. Amyloid-beta plaques indirectly affect GM volume, while tau tangles are regionally and tightly associated with GM volume reduction, leading to neurodegeneration [41]. GM atrophic patterns in the AD population may alter rTMS response because cortical current density is contingent on the type and extent of atrophy [42]. A correlation was found in a previous study [43] between GM atrophy in subjects with mild cognitive impairment (MCI) or AD and their changes in scores on the word part of the Stroop test after high-frequency (HF) rTMS of the superior temporal gyrus. After applying rTMS (five days/week) for four weeks, a previous study [44] did not find a substantial longitudinal difference in GM across six months between the active and sham intervention groups of patients with MCI.

The difference in WM volume or DLPFC asymmetry was insignificant in responders and non-responders in the active rTMS group at baseline, suggesting that WM volumetric patterns in the DLPFC are not predictors of treatment efficacy. However, both response groups showed rightward lateralization (left < right) in the WM of the DLFPC. Given that greater lateralization is related to declined cognitive abilities [33,45], this extreme lateralization in the WM of the DLPFC was expected at baseline in the two response groups. It is worth mentioning that we investigated these WM volumetric patterns using T1-weighted MRI data; however, T1 signal intensities are not sufficiently associated with WM integrity. Instead, fractional anisotropy with diffusion tensor imaging has been applied in the asymmetric pattern analysis of WM [30,46], and previous studies have also reported the impact of rTMS on alternations in WM fractional anisotropy in individuals with post-stroke aphasia and depression [47,48].

In the classification analysis of responders and non-responders to active treatment, our logistic regression model yielded an accuracy of 0.69 with an AUC of 0.74 using the GM volume in the right DLPFC and the asymmetry index of the DLPFC. These two GM features of the ROI could be used as predictive markers for rTMS efficacy, although the AUC was not in the range from 0.8 to 1, perhaps because of the small sample size.

The analysis within multiple regions throughout the brain did not yield significant differences in GM or WM between the responder and non-responder groups under active treatment. No region, including the DLPFC, reached the threshold for FWE correction (*p* < 0.05, with k > 50). A setting cut-off point of *p* < 0.05 for FWE correction seems strict in this study when our two groups of subjects had similar types of AD pathophysiology and might have subtle changes to be detected. Although FWE correction at threshold *p* < 0.05, a reference point, is highly recommended in neuroimaging research [49], several studies have also reported its stringent behavior in subtle lesion detection and instead suggested a liberal uncorrected threshold of *p* [50,51]. Supplementary Table S1 provides the whole-brain analysis results using an uncorrected *p* of 0.001.

In the correlation analysis, we noticed a significant negative correlation between the raw GM volumes of the left and right DLFPC and ADAS-Cog scores at the baseline of active treatment. This suggests that in general, the lower GM of the DLPFC increased the disease severity of our participants in the active treatment group. Such a significant correlation also exists in the right DLPFC when controlling for age, sex, and TIV. As noted in previous studies [9,52], a similar relationship between cognitive decline and GM volume also exists in other brain regions in people with AD/MCI.

When the data of the 12 participants in the sham group who received active treatment after the study period (6 months) were analyzed, the responders and non-responders (now 87 subjects) still showed a significant difference in the GM asymmetry index of the DLPFC. However, we ran the logistic regression only on the initial dataset (75 who were in the active rTMS group) for two main reasons: (1) the additional 12 subjects received active treatment in an open-label study, and (2) these 12 participants received active treatment 6–7 months after the baseline MRI.

This study has some limitations that should be considered when interpreting the results. First, the sample size was small, and the response group sizes were imbalanced. Second, this study obtained MRI scans from the participants at three sites scanned on different models of MRI scanners; although from the same manufacturer, a few scanning parameters differed from site to site. Third, the depression level of participants was not measured after treatment, and without having post-treatment measurements, we could not thoroughly investigate the effect of depression on MRI-driven features. Fourth, other factors, such as the distribution of CSF in the brain [53] and the degree and location of microvascular ischemic pathology, may act as confounding variables in treatment responses. Lastly, the lack of amyloid PET or fluid biomarkers to verify the amyloid status of these participants was a limitation of this study; it is also essential for future rTMS studies to include either imaging or fluid AD biomarkers to be sure of the participants’ biological diagnosis.

## 5. Conclusions

To the best of our knowledge, this study is the first to use volumetric measures of MRI data to predict rTMS treatment response for AD at baseline. GM volume in the right DLPFC or the asymmetry index in the GM of the DLPFC have shown potential, albeit weak, as predictive markers of the efficacy of active rTMS treatment. GM volumes in the DLPFC region were significantly associated with baseline ADAS-Cog scores of participants under active treatment.

## Figures and Tables

**Table 1:** Demographic and pretreatment baseline clinical data of responders and non-responders under active treatment.

	Responders	Non-Responders	Two-Tailed *p*
*n*	55	20	-
Sex (male, female)	32, 23	12, 8	0.902 ^†^
Age	72.5 ± 7.9	76.2 ± 5.7	0.055 ^††^
Handedness (left, right) *	2, 52	1, 19	0.680 ^†^
CSDD	4.3 ± 3.7	4.6 ± 2.6	0.362 ^‡^
MoCA	15.3 ± 5.2	16.2 ± 4.5	0.541 ^††^
CDR	1.1 ± 0.3	1.2 ± 0.4	0.535 ^‡^
ADAS-Cog	25.2 ± 9.3	20.8 ± 7.0	0.031 ^‡^

Vales are reported as mean ± SD. Cornell Scale for Depression in Dementia (CSDD), Montreal Cognitive Assessment (MoCA), Clinical Dementia Rating (CDR), and Alzheimer’s disease assessment scale—cognitive subscale (ADAS-Cog). * One responder had unknown handedness. ^†^ Two-proportion test. ^††^ Independent samples *t*-test. ^‡^ Wilcoxon rank-sum test.

**Table 2:** Gray matter (GM) and white matter (WM) volumes (cm^3^) in regions of interest for responders vs. non-responders of the active treatment group.

ROIs	Responders Mean ± SE	Non-Responders Mean ± SE	F * (1, 67)	*p* *
**GM**				
Left DLPFC	0.73 ± 0.02	0.72 ± 0.03	0.15	0.698
Right DLPFC	0.64 ± 0.02	0.72 ± 0.02	8.82	**0.004**
**WM**				
Left DLPFC	0.51 ± 0.02	0.51 ± 0.03	0.03	0.859
Right DLPFC	0.59 ± 0.02	0.57 ± 0.03	0.14	0.713

* ANCOVA statistics with covariates of age, sex, TIV, MRI site, and CSDD scores. The significance level was *p* < 0.05/2 = 0.025, following the Bonferroni correction for the comparison of two ROIs. DLPFC = dorsolateral prefrontal cortex; SE = standard error. The bold font of *p* values denotes a significant difference between responders and non-responders.

**Table 3:** Asymmetry index of the GM and WM in the DLPFC region for two response groups under active treatment.

Volumes	Responders Mean ± SE	Non-Responders Mean ± SE	F * (1, 67)	*p* *
GM	9.52 ± 0.86	5.06 ± 0.79	7.17	**0.009**
WM	9.24 ± 1.09	7.58 ± 1.39	0.99	0.324

* ANCOVA statistics with covariates of age, sex, TIV, MRI site, and CSDD scores. The bold font of *p* values denotes a significant difference between responders and non-responders.

**Table 4:** The logistic regression results for classifying responders and non-responders using significant MRI-driven features. Mean values of area under curve (AUC), sensitivity, specificity, and accuracy are presented.

Features	AUC	Sensitivity	Specificity	Accuracy
GM volume	0.71	0.67	0.62	0.65
Asymmetry index	0.70	0.63	0.72	0.66
GM volume and asymmetry index	0.74	0.65	0.77	0.69

## Data Availability

The data will be available upon a reasonable request from the PI of the study (ZM). Data are not yet available for the public due to ensuring all public data are fully de-identified.

## Supplementary Materials

The following supporting information can be downloaded at https://www.mdpi.com/article/10.3390/brainsci14030226/s1, Table S1: Whole-brain voxel-based morphometry (VBM) results using an uncorrected *p* of 0.001 to find the differences between responders and non-responders in gray matter (GM) and white matter (WM).
