# Supplementary material for: Can Brain Volume-Driven Characteristic Features Predict the Response of Alzheimer’s Patients to Repetitive Transcranial Magnetic Stimulation? A Pilot Study
Source: Brain Sci. 2024 Feb 28;14(3):226. doi: 10.3390/brainsci14030226 (PMC10968477; doi:10.3390/brainsci14030226)
Supplement: Supplementary file 1 [file brainsci-14-00226-s001.zip › brainsci-2886314-supplementary.pdf]

Supplementary Data:

We conducted a whole-brain voxel-based morphometry (VBM) on gray matter (GM) and white (WM) using a threshold of uncorrected p of 0.001 to find the differences between responders and non-responders. As shown in Supplementary Table 1, the responders had a lower GM volume in the right superior and middle frontal gyrus regions relative to non-responders. In contrast, the non-responders had a lower GM in the left occipital gyrus and right rectal gyrus than the responders. In the WM analysis, a trend of lower WM volume was in the left fusiform gyrus, left middle occipital gyrus, right calcarine fissure & surrounding cortex, and left inferior temporal gyrus of the responders than the non-responders. In the opposite contrast (responders > non-responders), no difference was observed in WM.

Supplementary Table S1: Whole-brain voxel-based morphometry (VBM) results using the uncorrected p of 0.001 to find the differences between responders and non-responders in gray matter (GM) and white (WM).

Extent	Voxel level-P_{uncorr}	T values	MNI coordinates (x, y, z) (mm)	Side	Regions
**GM: responders < non-responders**					
1253	0.000	4.60	22, 56, 20	R	Superior frontal gyrus
	0.000	4.06	22, 48, 22	R	Superior frontal gyrus
	0.001	3.38	32, 42, 18	R	Middle frontal gyrus
16	0.001	3.43	38, 26, 30	R	Middle frontal gyrus
**GM: responders > non-responders**					
131	0.000	3.56	−22, −88, 32	L	Superior occipital gyrus
9	0.001	3.27	4, 50, −26	R	Rectal gyrus
**WM: responders < non-responders**					
379	0.000	4.12	−40, −72, 4	L	Middle occipital gyrus
668	0.000	3.88	−34, −48, −6	L	Fusiform gyrus
	0.000	3.63	−42, −50, −14	L	Fusiform gyrus
	0.001	3.24	−36, -58, −10	L	Fusiform gyrus
52	0.000	3.55	2, −86, 14	R	Calcarine fissure & surrounding cortex
80	0.000	3.44	−34, −64, −14	L	Fusiform gyrus
35	0.001	3.39	−46, −34, −18	L	Inferior temporal gyrus
**WM: responders > non-responders**					
No differences					

MNI: Montreal Neurological Institute, L: left, R: right. Regions corresponding to the MNI coordinates are found in the xjView toolbox (https://www.alivelearn.net/xjview).
